# Long-term outcomes in primary membranous nephropathy: a Chinese cohort study with novel target antigen

**DOI:** 10.3389/fimmu.2026.1761515

**Published:** 2026-03-06

**Authors:** Ping Guo, Wenli Li, Shasha Chen, Xiangyu Yang, Lisha Ma, Changwei Wu, Guisen Li, Wei Wang

**Affiliations:** 1 School of Medicine, University of Electronic Science and Technology of China, Chengdu, China; 2 Renal Department and Nephrology Institute, Sichuan Provincial People’s Hospital, Chengdu, China

**Keywords:** long-term outcomes, NELL-1, PLA2R, primary membranous nephropathy (PMN), THSD7A

## Abstract

**Background:**

Long-term antigen-specific data in PMN among Chinese populations remain limited. This study evaluated six target antigens and their clinical significance during extended follow-up.

**Methods:**

We retrospectively analyzed 132 treatment-naïve PMN patients diagnosed by biopsy (2010–2018) and followed for a median of 62.9 months. Renal tissue expression of PLA2R, THSD7A, NELL-1, PCDH7, EXT1, and EXT2 was assessed by immunohistochemistry, and serum anti-PLA2R antibodies were measured by ELISA. Associations between antigen profiles and 5-year outcomes (remission, renal survival, malignancy) were evaluated.

**Results:**

PLA2R was the predominant antigen (84.1%), followed by THSD7A (5.3%) and NELL-1 (0.76%); no PCDH7, EXT1, or EXT2 positivity was detected. PLA2R-negative patients were more often female (71.4% vs. 36.0%, *P* = 0.003), with better renal function and more frequent C1q deposition (38.1% vs. 13.5%, *P* = 0.016). Serum anti-PLA2R antibodies were detected in 55.3% of patients and strongly correlated with tissue PLA2R positivity (AUC = 0.851; optimal cutoff ≥17.47 RU/mL). Baseline antibody titers were not associated with remission (*P* = 0.573). During 5-years follow-up, 42.4% achieved CR, 36.4% PR, and 21.2% had NR, with an estimated 5-year renal survival rate of 81.95%. No malignancy events were observed among the seven THSD7A-positive patients or the single NELL-1–positive patient in this cohort. Statistical power for rare antigen subgroups was limited.

**Conclusions:**

This >5-year Chinese PMN cohort provides the first comprehensive analysis of six target antigens. PLA2R remains predominant, while PLA2R-negative patients distinct immunopathologic features yet favorable long-term outcomes. A population-specific anti-PLA2R cutoff showed good diagnostic performance for predicting tissue antigen deposition. Rare antigens were infrequent and their malignancy associations require cautious interpretation. These findings provide long-term antigen-specific data supporting antigen-guided, population-adapted precision management of PMN.

## Introduction

Membranous nephropathy (MN), the primary cause of adult nephrotic syndrome, is characterized by subepithelial immune deposits, complement activation, proteinuria, and progressive renal impairment (1). Approximately 75% of cases are primary (PMN), driven by autoantibodies targeting podocyte antigens (1, 2). While the M-type phospholipase A2 receptor (PLA2R) is the dominant antigen in 70–80% of PMN cases (3), and thrombospondin type-1 domain-containing 7A (THSD7A) accounts for 1–5% of PLA2R-negative cases, about 20% of PMN patients lack detectable PLA2R or THSD7A (4). Recent discoveries of novel antigens - Neural Epidermal Growth Factor-Like 1 (NELL-1), protocadherin 7 (PCDH7), and exostosin 1/2 (EXT1/EXT2)-underscore the molecular heterogeneity of MN, encompassing both primary and secondary forms (5–8). Although numerous studies have established the short-term clinical course of PMN (typically 1–3 years), comprehensive data on long-term outcomes (>5 years) remain scarce, particularly in non-Western populations. While existing cohorts from Europe and North America report 5-year renal survival rates of 75%-89% (9, 10), these studies are largely limited to PLA2R and THSD7A antigen profiling. The prognostic significance of novel antigens such as NELL-1, PCDH7, and EXT1/2 over extended follow-up are poorly defined, and their distribution and clinical relevance in Chinese patients are scarcely documented. Given ethnic and regional variations in antigen prevalence and disease behavior, this gap warrants attention. Therefore, this study aimed to assess the long-term outcomes (>5 years) in a well-characterized Chinese PMN cohort with systematic evaluation of six target antigens (PLA2R, THSD7A, NELL-1, PCDH7, EXT1, and EXT2) in renal tissue and serum. By integrating antigen-specific data with extended clinical follow-up (median 62.9 months), we sought to clarify the clinicopathological correlates, remission patterns, renal survival, and malignancy risks across antigen subtypes, thereby addressing a significant research gap and contributing to antigen-guided management strategies in PMN.

## Patients and methods

### Study design and participants

This is a single-center observational, longitudinal retrospective cohort study. This retrospective study was approved by the Institutional Ethics Committee of Sichuan Provincial Hospital. Patients diagnosed with membranous nephropathy (MN) via renal biopsy (including light microscopy, immunofluorescence, and electron microscopy) at the Kidney Disease Center of Sichuan Provincial Hospital between May 1, 2010, and May 1, 2018, were initially screened. Exclusion criteria comprised: (1) Secondary MN (SMN) associated with autoimmune diseases, chronic hepatitis B virus infection, or other systemic disorders at diagnosis; (2) incomplete baseline data; and (3) estimated glomerular filtration rate (eGFR) <15 mL/min/1.73 m² at biopsy. A total of 132 treatment-naïve patients with complete baseline clinical, laboratory, and pathological data were included. Follow-up data were obtained through retrospective medical record review or telephone interviews. The follow-up period extended through December 31, 2022. Written informed consent was obtained from all participants for tissue and blood sample collection. The study was in accordance with the Declaration of Helsinki.

### Follow up

For each patient, the date of renal biopsy was considered the baseline. Follow-up was censored on December 31st, 2022. For patients not reaching this date, follow-up ended at the time of the last outpatient visit, patient death, or the onset of end-stage renal disease (ESRD) (defined as eGFR <15 mL/min/1.73 m² or initiation of renal replacement therapy). Clinical and laboratory data were collected retrospectively from patients’ electronic medical records and/or supplemented by telephone interviews at the following time points: baseline (biopsy date), 6 months after diagnosis, and then annually from the initial baseline measurement. Follow-up and Data Validation Structured telephone interviews were conducted by trained nephrology fellows to capture missing follow-up data. Using a standardized protocol, information was collected on renal outcomes (ESKD, dialysis), remission, adverse events (malignancy, infection), and mortality. Patient-reported data were verified against external medical records when accessible. Each 10–15 minute interview was performed by investigators blinded to antigen profiles. Ambiguous cases were adjudicated by a senior nephrologist to ensure data integrity and consistency.

A total of eight time points were evaluated for each patient within the study period (Presented as median with IQR (P25, P75) due to the non-normal distribution in retrospective studies with variable enrollment and endpoint timing.). The following parameters were systematically collected at each time point: Clinical Data such as Age at onset, Sex, Systolic blood pressure (SBP), Diastolic blood pressure (DBP), Diabetes history, BMI, White blood cell count (WBC), Hemoglobin (Hb), 24-hour urinary protein excretion, Blood urea nitrogen (BUN), Serum albumin, Serum creatinine, Estimated glomerular filtration rate (eGFR), Serum uric acid, Cystatin C, Total cholesterol, Triglycerides, Low-density lipoprotein cholesterol (LDL-C), High-density lipoprotein cholesterol (HDL-C), Serum IgG, Serum IgA, Serum IgM, Serum complement C3, Serum complement C4, Pathological Data such as Number of glomeruli, Proportion of interstitial fibrosis, Number of crescents, Immunofluorescence (IgG, IgM, IgA, C3, C1q, FRA), Morphological stage, Treatment regimen (e.g., immunosuppressive therapy), Remission status (complete/partial/no remission), Follow-up duration, Endpoints(Loss to follow-up/Death/ESRD), Safety(Treatment-related adverse events).

The median follow-up was 62.9 months (IQR 32.4-87.4 months).

### Outcome and definitions

This study used composite endpoints to assess disease progression and treatment response. The primary outcomes include renal survival and remission status.

Renal Survival was defined as the first occurrence of composite endpoint of the following events during follow-up: 1) A decline in estimated glomerular filtration rate (eGFR) of ≥40% from baseline over at least 4 weeks (11). 2)Progression to end-stage renal disease (ESRD, eGFR <15 mL/min/1.73 m² or requiring renal replacement therapy).3) Death from renal causes. Time-to-event was calculated from the date of renal biopsy to the occurrence of the composite endpoint or censoring at the last available follow-up. Remission criteria during follow-up were defined according to the 2021 KDIGO guidelines (12), with primary monitoring of three parameters: urinary protein excretion, serum albumin, and serum creatinine.

Secondary outcomes include changes in proteinuria, dynamics of serological markers, histological progression, and safety endpoints (incidence of malignancies, severe infectious events, and adverse reactions related to immunosuppressants), etc.

In the biopsy study, the Ehrenreich-Churg histological classification system was used (13).

### Antigen detection

Immunohistochemical (IHC) staining for renal tissue antigen profiling was performed on 2-μm formalin-fixed paraffin-embedded sections using standardized protocols. Following deparaffinization in xylene and rehydration through graded ethanol, antigen retrieval was conducted in preheated citrate buffer (pH 6.0) under high-pressure conditions (120 °C) for 3 minutes. Endogenous peroxidase activity was blocked with 3% H_2_O_2_ (30 min, RT). Sections were then incubated overnight at 4 °C with specific antibodies against PLA2R, THSD7A, NELL-1, PCDH7, EXT1, and EXT2. After PBS washes, HRP-conjugated goat anti-rabbit/mouse IgG polymer (1:200) was applied for 30 min at RT. Chromogenic development used DAB substrate with hematoxylin counterstaining. Positive staining was defined as granular dark-brown deposits along glomerular basement membranes. Negative controls used PBS instead of primary antibody. All primary antibodies used in this study, including those targeting low-prevalence antigens such as PCDH7, EXT1, and EXT2, have been previously established to reliably detect their respective targets in human renal tissues via immunohistochemistry (IHC) or mass spectrometry–guided proteomic approaches.

Due to the retrospective nature of this study and the limited accessibility of confirmed antigen-positive renal biopsy specimens for rare subtypes, dedicated in-run positive control sections for PCDH7, EXT1, and EXT2 were not included. However, to ensure methodological rigor and exclude non-specific staining, negative controls—conducted by omitting the primary antibodies—were performed in parallel for all staining procedures (**Supplementary Figures 1–3**). All immunohistochemical slides were independently reviewed by two experienced renal pathologists who were blinded to clinical data. Discrepancies were resolved by joint review to reach consensus.

Serum Anti-PLA2R Antibody Detection: Serum anti-PLA2R antibody levels were measured using fasting blood samples (approximately 5 mL) collected from patients at the time of clinical diagnosis, prior to kidney biopsy. After collection according to routine hospital laboratory procedures, samples were centrifuged and stored at -80 °C until being transported to Shanghai Xinpeijing Medical Laboratory for analysis. Quantitative measurement was performed using a commercially available enzyme-linked immunosorbent assay (ELISA) kit (Anti-PLA2R IgG ELISA, EUROIMMUN, Lübeck, Germany), strictly following the manufacturer’s instructions. Results were expressed in relative units per milliliter (RU/mL), derived from a manufacturer-provided calibrator based on internal reference sera. Anti-PLA2R antibody levels <14 RU/mL are considered negative, 14–<20 RU/mL borderline, and ≥20 RU/mL positive. It should be noted that these RU/mL values represent semi-quantitative, platform-specific measurements; therefore, they are not directly interchangeable across different assay systems or manufacturers.

### Statistical analysis

Data were analyzed using SPSS 26.0 and R version 4.5.1. Continuous variables with normal distribution are expressed as mean ± SD (Student’s t-test), while non-normally distributed variables are reported as median (P25, P75) (Mann-Whitney U test). Categorical variables are presented as percentages (Pearson’s χ² test or Fisher’s exact test). Correlations were evaluated using Pearson’s method; survival differences were assessed via Kaplan-Meier analysis. with the log-rank test employed to compare survival distributions between cohorts. To identify independent factors associated with renal outcomes, univariable and multivariable Cox proportional hazards regression models were constructed. To evaluate the predictive performance of identified risk factors, we calculated the area under the receiver operating characteristic (ROC) curve (AUC). The robustness of the renal survival estimates was further validated through a sensitivity analysis using inverse probability of censoring weighting (IPCW) to mitigate potential bias arising from informative censoring. No deaths or non-renal events occurred within 5 years, so competing-risk analyses were omitted. Statistical significance was defined as P < 0.05.

### Sample size and power analysis

This study was retrospective in design, and the sample size was determined by the number of eligible patients identified during the study period. Therefore, no formal *a priori* sample size calculation was performed. A *post-hoc* power analysis was conducted using PASS version 15.0 to evaluate the sensitivity of the study. The primary endpoint was defined as renal progression (≥40% decline in eGFR or progression to ESRD). Based on the observed cohort size (n = 132) and the total number of renal events (n = 31), the estimated statistical power was approximately 80% to detect a hazard ratio (HR) of 2.0 between PLA2R-positive and PLA2R-negative groups, assuming a two-sided α level of 0.05. For a more conservative effect size (HR = 1.5), the estimated power decreased to approximately 60–65%, indicating adequate sensitivity for detecting moderate-to-large associations, but limited power for identifying small effect sizes. Importantly, no meaningful power estimation could be performed for rare antigen subgroups (THSD7A, NELL-1) due to extremely small sample sizes.

## Results

**Figure 1:**
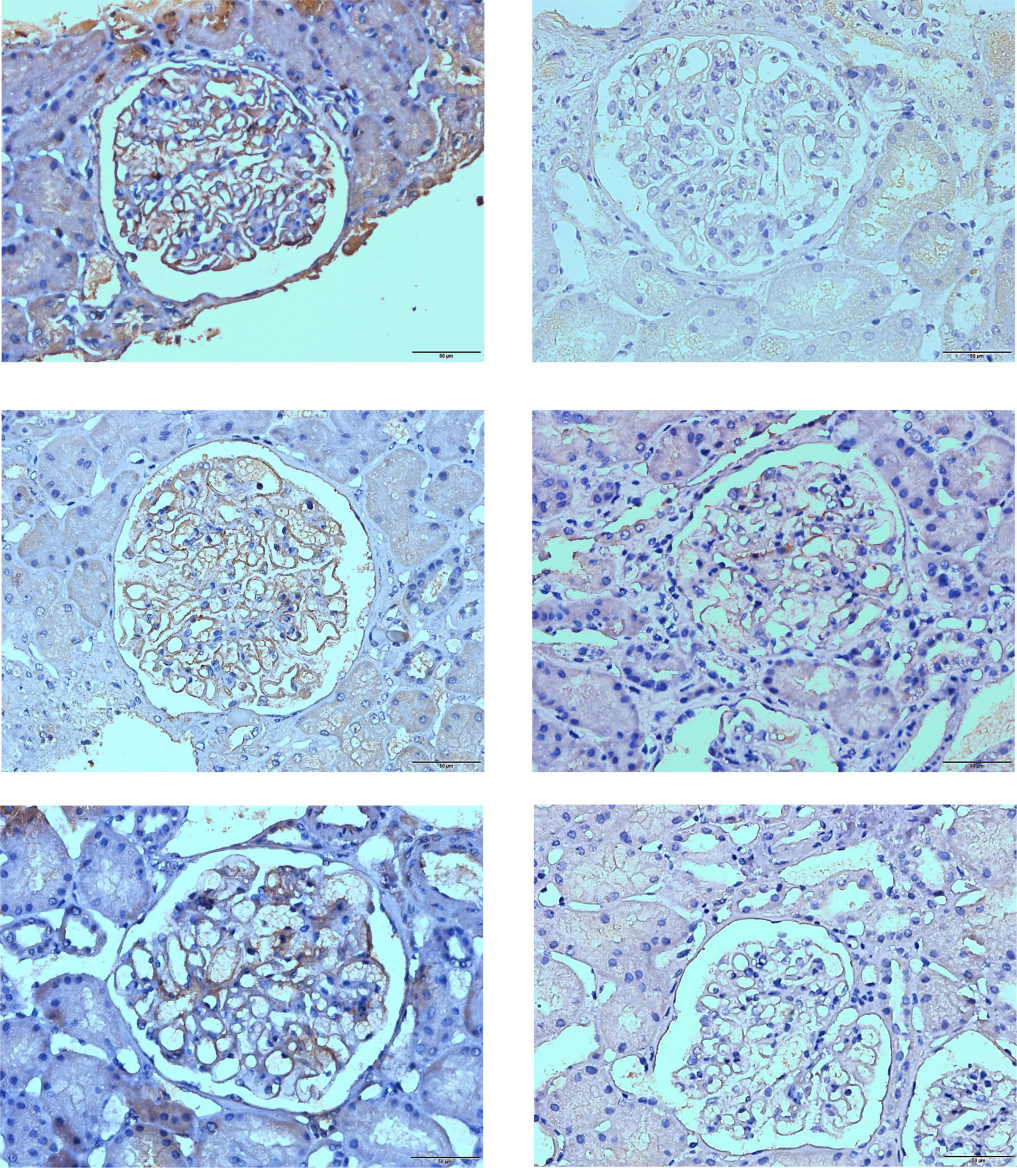
Representative immunohistochemical (IHC) staining for PLA2R, THSD7A, and NELL-1 is shown. **(A)** Positive immunohistochemical staining for PLA2R; **(B)** Negative control for PLA2R. **(C)** Positive immunohistochemical staining for THSD7A; **(D)** Negative control for THSD7A. **(E)** Positive immunohistochemical staining for NELL-1; **(F)** Negative control for NELL-1. Original magnification: ×400.

**Figure 2:**
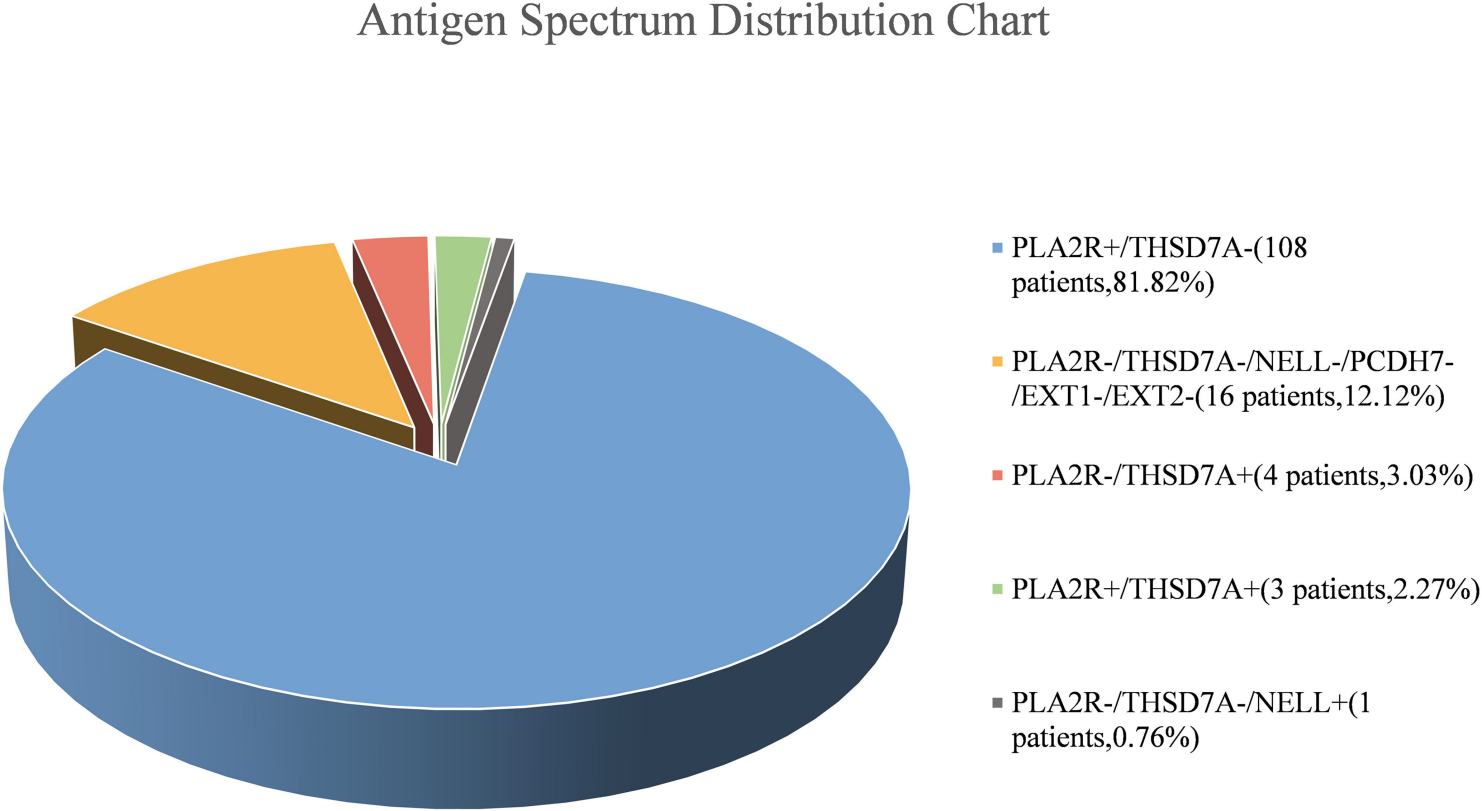
Distribution of target antigen profiles (n=132).

This study represents a comprehensive screening for six target antigens (PLA2R, THSD7A, NELL-1, PCDH7, EXT1, and EXT2) in a Chinese cohort 132 treatment-naïve patients with PMN. Immunohistochemistry (IHC) was performed on kidney tissue specimens from all 132 PMN patients. PLA2R was the predominant antigen, detected in 84.1% (111/132) of cases [**Figures 1A, B**]. THSD7A staining was observed in 5.3% (7/132), including 3 patients with concurrent PLA2R positivity [**Figures 1C, D**]. Within the 17 PLA2R/THSD7A double-negative cases, one NELL-1-positive case was identified (5.9% of double-negative patients; 0.76% of the total cohort) [**Figures 1E, F**]. Notably, no positive staining for PCDH7, EXT1, or EXT2 antigens was detected in any patient [**Supplementary Figures 1**-**3**], these subgroups were excluded from the subsequent long-term outcome analyses. The comprehensive antigenic profile distribution within the cohort is illustrated in **Figure 2**. At baseline, anti-PLA2R antibodies were detected in the serum of 55.3% (73/132) of patients. Comprehensive analyses were performed to evaluate the clinical characteristics and long-term outcomes associated with each antigen. The extended follow-up duration (median: 62.9 months; IQR: 32.4–87.4 months) provided a robust foundation for assessing longitudinal outcomes and yielded critical long-term prognostic data, including a 5-year renal survival rate of 81.95% for PMN patients.

### Expression and clinical significance of PLA2R antigen in renal tissue of PMN

Immunohistochemical (IHC) staining for PLA2R was performed on renal tissues from 132 PMN patients, with 111 cases (84.1%) classified as PLA2R-positive and 21 cases (15.9%) as PLA2R-negative. In terms of baseline clinical characteristics, significant differences were noted in sex distribution, serum creatinine, eGFR, and serum IgG levels (P<0.05). The PLA2R-negative group exhibited a higher proportion of females (71.4% vs. 36.0%, P = 0.003), and kidney function is better, with lower serum creatinine levels (median: 78.5 vs. 92.0 μmol/L, P = 0.002) and higher eGFR (median: 85.3 vs. 72.1 mL/min/1.73 m², P = 0.048). Additionally, the serum IgG levels is higher (median: 12.4 vs. 9.8 g/L, P = 0.034) (**Table 1**). It’s worth noting that in terms of pathological features, C1q deposition was more frequent in the PLA2R-negative group (38.1% vs. 13.5%, P = 0.016), with no differences observed in other pathological indicators (**Table 2**). In terms of treatment and prognosis, no significant differences were observed in immunosuppressive treatment regimens or clinical outcomes between the two groups (*P* > 0.05) (**Table 3**).

**Table 1:** Comparison of baseline clinical characteristics between PLA2R-positive and PLA2R-negative groups.

Parameter	PLA2R-positive group (n=111)	PLA2R-negative group (n =21)	P-value
Age (years)	52 (44-60)	49 (42-61)	0.690
Sex,n (%)			0.003*
Male	71 (64.0)	6 (28.6)	
Female	40 (36.0)	15 (71.4)	
Systolic BP (mmHg)	135 (121-147)	137 (123.5-148)	0.911
Diastolic BP (mmHg)	81 (72-89)	82 (75-91)	0.723
Diabetes,n (%)	14 (12.6)	1 (4.8)	0.506
BMI (kg/m2)	24.1 (22.2-26.0)	24.0 (22.4-26.0)	0.963
WBC (109/L)	6.8 (5.6-8.6)	6.6 (5.5-9.0)	0.926
Hemoglobin (g/L)	134 (122-145)	130 (126.5-141.5)	0.917
24-hour urinary protein (g/24h)	5.3 (3.4-8.6)	4.1 (2.4-6.7)	0.090
BUN (mmol/L)	5.3 (4.1-6.7)	4.7 (3.4-6.4)	0.210
Serum albumin (g/L)	23.3 (19.9-28.3)	25.9 (21.2-31.0)	0.140
Serum creatinine (μmol/L)	70.9 (55.5-82.1)	50.2 (44-70.9)	0.002*
eGFR (mL/min/1.73 m2)	99.8 (87.4-110.0)	109.2 (92.1-116.3)	0.048*
Uric acid (μmol/L)	355.0 (305.0-428.0)	328.3 (260.4-390.0)	0.107
Cystatin C (mg/L)	1.0 (0.9-1.4)	0.9 (0.7-1.4)	0.163
Total cholesterol (mmol/L)	7.2 (6.3-8.9)	7.6 (5.6-8.9)	0.772
Triglycerides (mmol/L)	2.3 (1.7-3.3)	1.6 (1.3-3.1)	0.109
LDL-C (mmol/L)	4.2 (3.4-5.7)	4.6 (3.1-5.9)	0.109
HDL-C (mmol/L)	1.7 (1.2-2.0)	1.7 (1.3-2.2)	0.723
Serum IgG (g/L)	5.5 (4.3-7.2)	7.1 (4.9-11.6)	0.034*
Serum IgA (g/L)	2.1 (1.5-2.7)	2.4 (1.7-3.5)	0.208
Serum IgM (g/L)	1.4 (0.9-2.0)	1.4 (1.0-2.2)	0.586
Complement C3 (g/L)	1.1 (0.9-1.4)	1.2 (1.0-1.3)	0.845
Complement C4 (g/L)	0.3 (0.2-0.4)	0.3 (0.2-0.4)	0.150

* indicates P < 0.05.

**Table 2:** Comparison of pathological features between PLA2R-positive and PLA2R-negative groups.

Parameter	PLA2R-positive group (n=111)	PLA2R-positive group (n =21)	P-value
Number of glomeruli (n)	12 (8-17)	13 (9-16)	0.609
Interstitial fibrosis, n (%)			1.000
<25%	109 (98.2)	21 (100)	
25%-50%	2 (1.8)	0	
>50%	0	0	
Crescents, n (%)	6 (5.4)	1 (4.8)	1.000
Immunofluorescence deposits, n (%)			
IgG	111 (100)	20 (95.2)	0.159
IgM	27 (24.3)	5 (23.8)	0.960
IgA	20 (18.0)	3 (14.2)	0.920
C3	97 (87.4)	16 (76.2)	0.317
C1q	15 (13.5)	8 (38.1)	0.016*
FRA	8 (7.2)	0	0.441
Morphological stage, n (%)			0.492
Stage I and I–II	82 (73.9)	17 (81.0)	
Stage II and II–III	29 (26.1)	4 (19.0)	
Stage III and III–IV	0	0	

* indicates P < 0.05.

**Table 3:** Comparison of treatment and prognosis between PLA2R-positive and PLA2R-negative groups.

Parameter	PLA2R-positive group (n=111)	PLA2R-positive group (n =21)	P-value
Treatment, n (%)			0.523
Immunosuppressive therapy	94 (84.7%)	16 (76.2%)	
Non-immunosuppressive therapy	17 (15.3%)	5 (23.8%)	
Prognosis, n (%)			0.102
No remission	26 (23.4%, 95% CI: 16.7-33.4%))	1 (4.8%, 95% CI: 0.1-23.8%))	
Partial remission	38 (34.2%, 95% CI: 24.7-42.9%)	11 (52.4%, 95% CI: 29.8-74.3%)	
Complete remission	47 (42.3%, 95% CI: 33.0-52.1%)	9 (42.9%, 95% CI: 21.8-66.0%))	
Follow-up duration (months)	64.4 (32.4-87.4)	48.9 (23.9-92.5)	0.524

### Detection and clinical significance of serum anti-PLA2R antibodies in PMN

Serum samples were collected from 132 treatment-naïve PMN patients at disease onset for anti-PLA2R antibody detection. In terms of baseline clinical characteristics and pathological features, serum anti-PLA2R antibody positivity showed positive correlations with 24-hour urinary protein excretion (*r* = 0.259, *P* = 0.003), total cholesterol (*P* < 0.05), triglycerides (*P* < 0.05), low-density lipoprotein cholesterol (*P* < 0.05), serum complement C4 (*P* < 0.05), immunofluorescence C3 intensity (*P* < 0.05), and advanced morphological stage (*P* < 0.05). Negative correlations were observed with hemoglobin (*P* < 0.05), serum albumin (*P* < 0.05), and serum IgG (*P* < 0.05) (**Table 4**).

**Table 4:** Correlation analysis between serum anti-PLA2R antibody and clinical/pathological parameters.

Parameter	Serum anti-PLA2R antibody	
	Correlation coefficient (r)	P-value
Age(years)	0.055	0.355
Hemoglobin (g/L)	-0.195	0.025*
24-hour urinary protein excretion (g/24h)	0.259	0.003*
BUN (mmol/L)	0.056	0.522
Serum albumin (g/L)	-0.363	<0.01*
Serum creatinine (μmol/L)	0.169	0.052
eGFR*(mL/min/1.73m2)	-0.165	0.058
Uric acid (μmol/L)	-0.124	0.157
Cystatin C (mg/L)	0.106	0.226
Total cholesterol (mmol/L)	0.223	0.010*
Triglycerides (mmol/L)	0.279	0.001*
LDL-C (mmol/L)	0.174	0.046*
HDL-C (mmol/L)	-0.119	0.173
Serum IgG(g/L)	-0.283	0.001*
Serum IgA(g/L)	0.006	0.942
Complement C3(g/L)	0.116	0.186
Complement C4(g/L)	0.216	0.013*
Proportion of interstitial fibrosis (%)	-0.052	0.467
Immunofluorescence IgG intensity	0.104	0.149
Immunofluorescence IgM intensity	-0.078	0.280
Immunofluorescence IgA intensity	-0.018	0.806
Immunofluorescence C3 intensity	0.161	0.025*
Immunofluorescence C1q intensity	-0.055	0.444
Immunofluorescence FRA intensity	0.038	0.600
Morphological stage	0.155	0.031*

* indicates P < 0.05.

**Figure 3:**
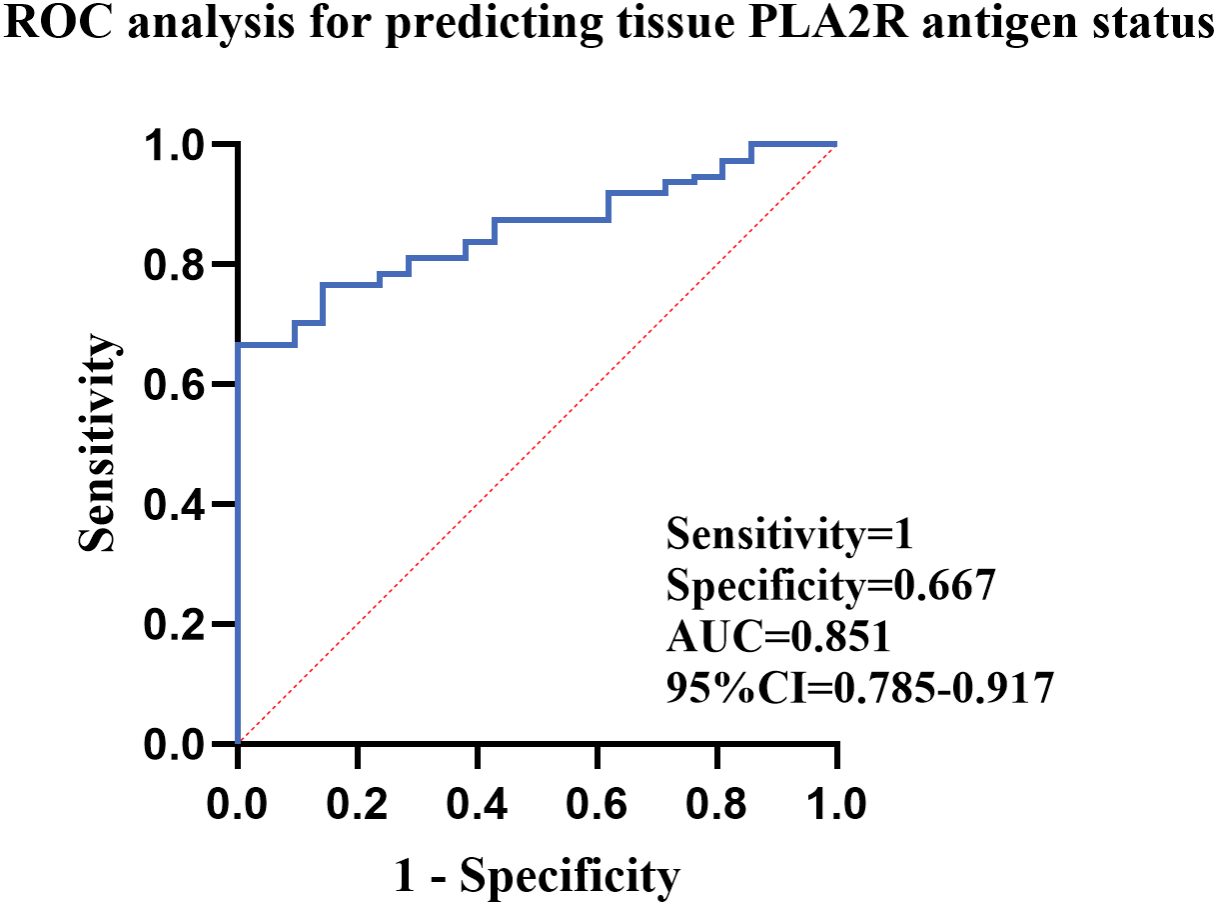
Correlation analysis between serum anti-PLA2R antibodies and renal tissue PLA2R antigen expression.

Serum anti-PLA2R antibodies were positive in 73 patients (55.3%). The findings were highly consistent with renal tissue PLA2R deposition (χ²=30.899, P<0.001): 65.8% of tissue-positive cases showed serum positivity, while 100% of tissue-negative cases were serum-negative (**Table 5**). The receiver operating characteristic (ROC) curve demonstrated that serum anti-PLA2R antibodies predicted renal tissue PLA2R antigen deposition with an area under the curve(AUC) of 0.851 (95% CI: 0.785–0.917; P < 0.001), with an optimal cut-off value ≥17.47 RU/mL defined by the maximum Youden index [**Figure 3**].

**Table 5:** Serum anti-PLA2R antibody expression in patients with primary membranous nephropathy.

Renal tissue PLA2R antigen	Serum anti-PLA2R antibody		Total	χ²	P-value
	Positive (n=73)	Negative (n=59)			
Positive (n=111)	73 (65.8%, 95%CI: 56.2-74.5%)	38 (34.2%, 95%CI: 25.5-43.8%)	111 (84.1%)	30.899	<0.001*
Negative (n=21)	0 (0.0%)	21 (100.0%, 95%CI: 83.9-100.0%)	21 (15.9%)		
Total	73 (55.3%)	59 (44.7%)	132		

* indicates P < 0.05.

**Figure 4:**
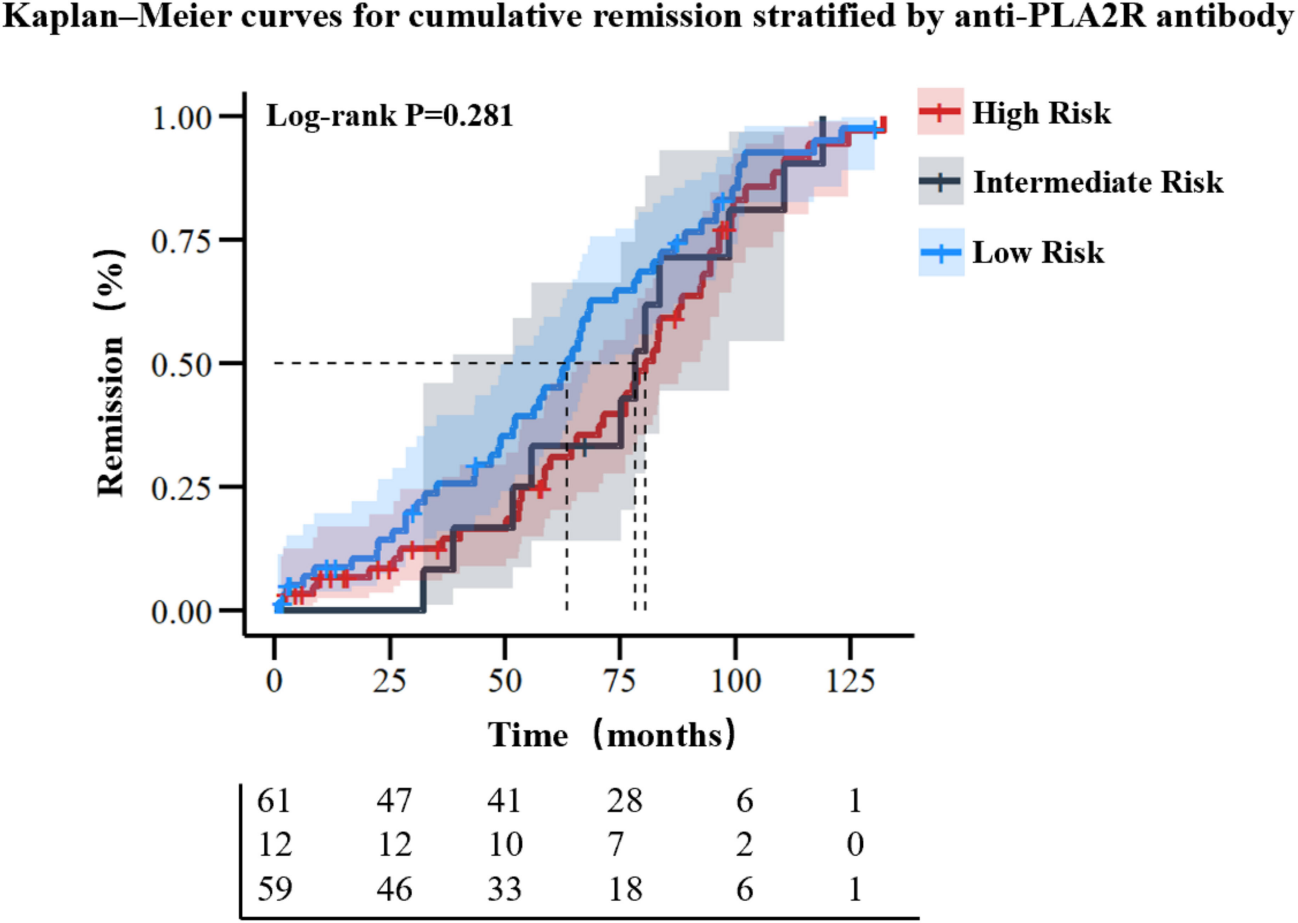
Kaplan-Meier analysis of cumulative remission rates stratified by serum anti-PLA2R antibody titer levels.

To evaluate the prognostic impact of baseline anti-PLA2R antibody levels, patients were stratified into low- (<20 RU/mL), intermediate- (20–50 RU/mL), and high-risk (>50 RU/mL) groups based on 2021 KDIGO risk classification (12). Over a median follow-up of 62.9 months (range: 32.4-87.4 months), no significant differences in remission rates were observed among the three groups (*P* = 0.573) (**Table 6**). Kaplan-Meier analysis with right-censoring (to account for variable follow-up durations: low-risk group median 57.4 months vs. intermediate-risk 76.6 months) revealed no statistically significant differences in cumulative remission rates among the three groups (log-rank test: χ² = 2.542, P = 0.281) [**Figure 4**].

**Table 6:** Comparison of remission rates across serum anti-PLA2R antibody titer levels.

Anti-PLA2R antibody titer (RU/mL)	Follow-up duration (months)	No remission, n (%)	Partial remission, n (%)	Complete remission, n (%)
<20	57.4(28.3-83.8)	10(16.9%,95%CI: 8.4-29.0%)	23(39.0%, 95%CI: 26.5-52.6%)	26(44.1%, 95%CI: 31.2-57.6%)
20-50	76.6(52.6-94.8)	1(8.3%, 95%CI: 0.2-38.5%)	6(50.0%, 95%CI: 21.1-78.9%)	5(41.7%, 95%CI: 15.2-72.3%)
>50	65.5(26.6-92.7)	16(26.2%, 95%CI: 17.1-40.8%)	20(32.8%, 95%CI: 19.9-44.3%)	25(41.0%, 95%CI: 28.6-54.3%)
P-value	0.22		0.573	

Intergroup comparisons were performed using the chi-square test. After Bonferroni correction (adjusted significance level α = 0.05/3 = 0.0167 for three-group comparisons).

### Expression and clinical characteristics of THSD7A in renal tissue of PMN

Immunohistochemical (IHC) staining for THSD7A was performed on renal tissues from 132 PMN patients. THSD7A positivity was detected in 7 cases (5.3%). Among these, renal tissue was PLA2R-positive in 3 patients, and serum anti-PLA2R antibodies were positive in 2 patients. In these 7 THSD7A-positive patients (4 male, 3 female), the median age at disease onset was higher (70 years [IQR: 68–74]). Renal function was normal at onset (serum creatinine: median 71.4 μmol/L [IQR: 59.5–78.9]). During a median follow-up of 55.9 months (IQR: 43.63–99.53), six patients received immunosuppressive therapy (cyclophosphamide), with 5 achieving complete remission, and one patient received non-immunosuppressive treatment (RAS inhibition) and did not achieve remission. No malignancies event occurred in the seven patients during follow-up [**Table 7**].

**Table 7:** Clinical characteristics of THSD7A-positive PMN patients.

Parameter	Case 1	Case 2	Case 3	Case 4	Case 5	Case 6	Case 7	Median (IQR)
Age (years)	70	65	74	77	68	74	69	70 (68–74)
Gender	male	female	male	male	female	female	male	
Renal tissue PLA2R staining	+	–	–	+	–	+	–	
Serum anti-PLA2R antibody	+	–	–	–	–	+	–	
24-hour urinary protein (g/24h)	6.65	2.24	4.12	3.67	5.20	6.11	2.75	4.12 (2.75–6.11)
Serum albumin (g/L)	16.7	23.8	22	20.5	28.1	26.7	20.4	22.0 (20.5–26.7)
Serum creatinine (μmol/L)	71.4	45	111	78.9	59.5	65.5	126.6	71.4 (59.5–78.9)
eGFR (mL/min/1.73m²)	90.2	101	56.1	82.4	90.2	79.7	49.5	82.4 (56.1–90.2)
Glomeruli/sclerosed glomeruli	40/0	13/0	8/1	6/0	16/0	18/0	10/2	13/0 (8/0–16/0)
Immunofluorescence (Intensity)	IgG (3+) IgA (2+) C3 (2+)	IgG (3+) C3 (2+) C1q (1+)	IgG (3+) C3 (3+) C1q (1+)	IgG (2+) IgM (1+) C3 (3+)	IgG (2+) C3 (3+)	IgG (3+) C3(2+)	IgG (2+) IgM (1+) IgA (1+) C3 (2+)	
Morphological Stage	I-II	I	II	I	I	I-II	I-II	
Comorbidities	None	None	None	None	None	None	None	
Treatment Regimen	Corticosteroids + CNI	Non-immunosuppressive therapy	Corticosteroids + CNI	Corticosteroids	Corticosteroids + CTX	Corticosteroids + CTX	Corticosteroids + CTX	
Remission Status	CR	CR	NR	CR	CR	CR	PR	
Follow-up Duration (months)	94.53	100.6	43.63	68	123.067	99.53	16.8	55.9(43.63–99.53)

eGFR, estimated glomerular filtration rate, calculated using the CKD-EPI (Chronic Kidney Disease Epidemiology Collaboration) equation; CR, complete remission; PR, partial remission; NR, no remission; CTX, cyclophosphamide; CNI, calcineurin inhibitor.

### Expression and clinical evaluation of NELL-1 in renal tissue of PMN

Among 17 PLA2R/THSD7A double-negative PMN patients, renal tissues were further evaluated for NELL-1 expression via IHC staining, with positivity identified in 1 case (5.9%). The NELL-1-associated PMN patient was a 48-year-old female (**Table 8**). At diagnosis, she presented with hypoalbuminemia (serum albumin: 23.2 g/L), nephrotic-range proteinuria (3.54 g/24h), and preserved renal function (serum creatinine and eGFR within normal ranges). Renal biopsy revealed IgG++ and C3+++ deposits by immunofluorescence, no sclerosed glomeruli on light microscopy, and morphological stage I (Ehrenreich-Churg classification). The patient received immunosuppressive therapy with corticosteroids combined with cyclophosphamide. Proteinuria decreased to 0.075 g/24h at 6 months, achieving CR. Notably, no malignancy or other secondary causes emerged during this long-term follow-up (78.1 months) in this NELL-1-positive case.

**Table 8:** Clinical characteristics of the NELL-1-associated membranous nephropathy patient.

Target antigen	Age(years)	Gender	Serum Creatinine (μmol/L)	eGFR (mL/min/1.73m²)	Glomeruli/sclerosed glomeruli	Immunofluorescence (Intensity)	Comorbidities	Treatment regimen	Remission status	Follow-up (months)
NELL-1	48	female	54	107.2	13/0	IgG(2+) C3(3+)	None	Corticosteroids + CTX	CR	78.1

eGFR, estimated glomerular filtration rate, calculated using the CKD-EPI (Chronic Kidney Disease Epidemiology Collaboration) equation; CR, complete remission; CTX, cyclophosphamide.

Notably, none of the 132 patients showed positive staining for EXT1, EXT2, or PCDH7.

### Long-term outcomes over 5 years

Among 132 treatment-naïve PMN patients followed for a median of 62.9 months (IQR: 32.4–87.4), 31 composite renal endpoint events occurred. All 31 patients reached the threshold of a ≥40% decline in eGFR from baseline, and among them, 6 patients eventually progressed to ESRD. Thirty-eight patients were censored due to loss to follow-up or a follow-up duration of less than five years; notably, all censored patients remained alive and free of renal events at their last documented visit. Based on the Kaplan–Meier analysis, the estimated 5-year renal survival rate was 81.95% (95% CI: 75.17%–89.33%). To assess the potential impact of censoring on renal survival analyses, baseline characteristics were compared between censored and non-censored patients. No clinically meaningful or statistically significant imbalances were observed between the two groups (**Supplementary Table 1**). Although univariable log-rank analysis suggested a difference in renal survival, multivariable Cox regression adjusting for age, baseline eGFR, and anti-PLA2R antibody status demonstrated that censoring status was not an independent predictor of renal survival (**Supplementary Table 2**).

**Figure 5:**
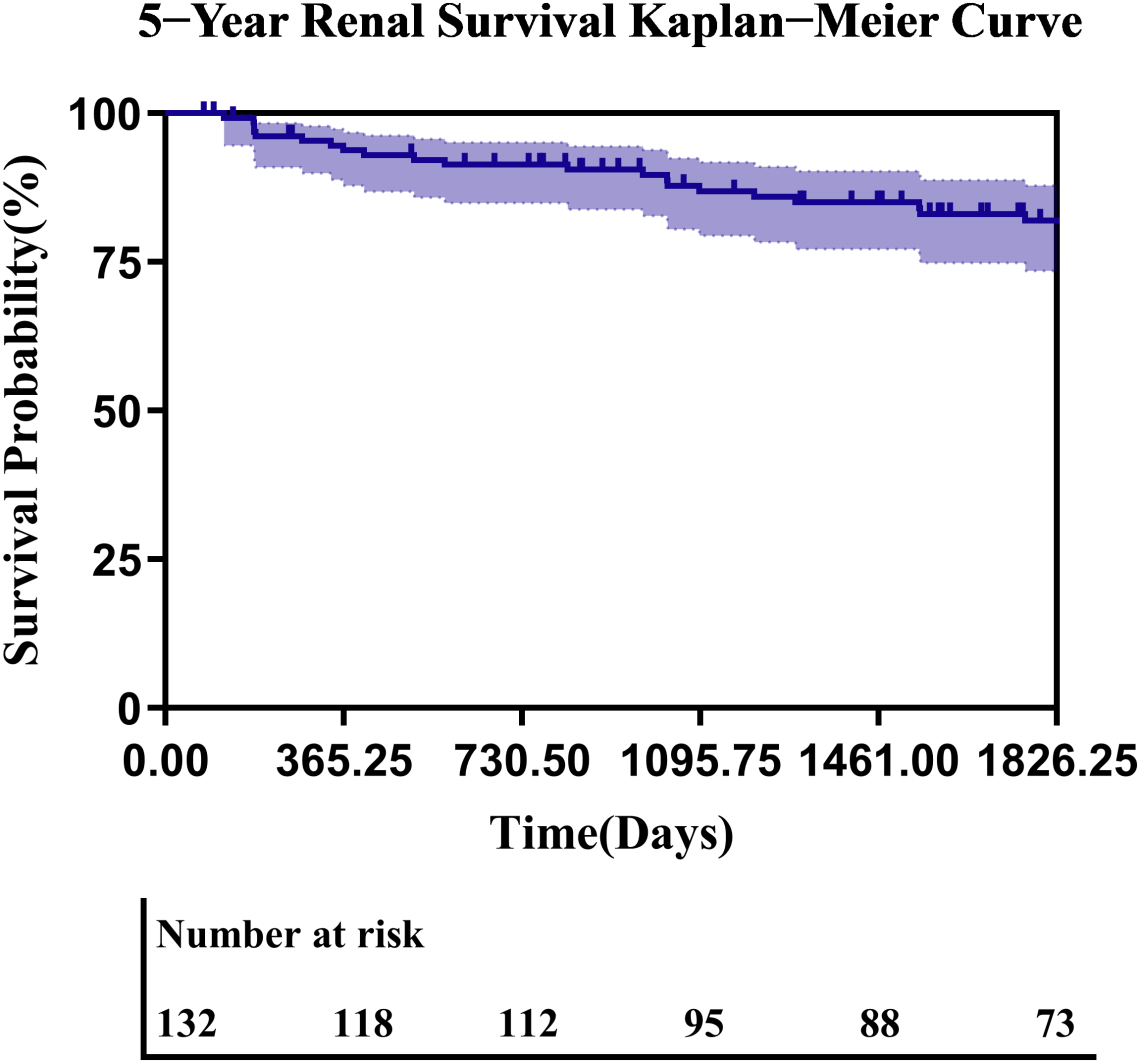
5-year renal survival Kaplan−Meier curve.

The renal survival curve remained relatively stable following the initial follow-up period (**Figure 5**). In multivariable Cox regression analysis adjusting for age, sex, baseline eGFR, baseline proteinuria, immunosuppressive therapy, and C1q deposition, only baseline proteinuria remained an independent predictor of renal outcome (adjusted HR 1.28 per 1 g/24h increase, P < 0.001), whereas PLA2R antigen positivity was not independently associated with renal survival (adjusted HR 2.29, 95% CI 0.61–8.70; **Supplementary Table 3**). In addition, inverse probability of censoring weighting (IPCW) analysis was performed as a sensitivity analysis to further evaluate the robustness of renal survival estimates. The IPCW-adjusted 5-year renal survival rate differed minimally from the primary Kaplan–Meier estimate (absolute difference: 2.28%), indicating that censoring introduced only weak informative bias and had a limited impact on the primary conclusions (**Supplementary Figure 4**). Regarding treatment response within the 5-year follow-up period, 42.4% (56/132) of patients achieved CR, 36.4% (48/132) achieved partial PR, and 21.2% (28/132) exhibited no remission. No significant difference in 5-year remission rates was observed between PLA2R^+^ (84%) and PLA2R^-^ (16%) subgroups (P = 0.33, log-rank test; **Supplementary Figure 5**). Notably, all seven THSD7A^+^ patients achieved CR. The single NELL-1^+^ patient maintained sustained CR without disease progression throughout follow-up. Antigen subtype (PLA2R^+^/^-^ or THSD7A^+^) did not significantly impact renal outcomes(P>0.05). Both THSD7A^+^ and NELL-1^+^ patients, along with other reported high-risk groups, did not develop malignancies or other triggering factors during the follow-up period.

## Discussion

This study is the first to systematically analyze the prevalence, clinicopathologic features, and long-term outcomes of six target antigens—PLA2R, THSD7A, NELL-1, PCDH7, EXT1, and EXT2—in a Chinese PMN cohort with a follow-up period exceeding 5 years (median duration, 62.9 months). It provides population-specific data on antigen distribution and clinical significance in Chinese PMN. Our main findings indicate that PLA2R is the predominant antigen (84.1%). PLA2R-negative cases were characterized by a higher proportion of females, better baseline renal function, and more frequent C1q deposition. The seropositivity rate of anti-PLA2R antibodies was 55.3%, and an antibody level ≥17.47 RU/mL showed good diagnostic performance in predicting tissue PLA2R positivity (AUC = 0.851). However, antibody titers were not significantly associated with remission rates (P = 0.573). Overall, antigen subtypes did not differ significantly in terms of 5-year renal outcomes, with a 5-year renal survival rate of approximately 81.95%. These findings highlight the value of antigen-driven stratification in delineating disease heterogeneity and refining prognostic assessment in PMN, thereby broadening our understanding of antigenic diversity in this condition.

Compared with previous reports, the tissue PLA2R positivity rate in our cohort was slightly lower than that reported in other Asian cohorts (84.1% vs. 95.6% in Pang et al. (14)) but higher than that observed in Western cohorts (70–80%) (2). The seropositivity rate of anti-PLA2R antibodies was consistent with that reported in Asian populations (55.3% vs. 58.8% (14)), yet lower than that in Western populations (70%) (2). These findings clearly reflect the racial differences in antigen distribution as well as the heterogeneity associated with detection methods and disease stages, while further reinforcing the central diagnostic role of PLA2R in this population.

Interestingly, we observed a higher rate of tissue PLA2R antigen positivity (84.1%) compared with serum antibody positivity (55.3%), which is consistent with findings from other cohort studies (14–18). This discrepancy may reflect local antibody production, in which anti-PLA2R antibodies originate from glomerular antigen exposure. Circulating antibodies exhibit high-affinity binding to PLA2R antigens within the glomeruli, and detectable serum antibodies appear only after saturation of glomerular antigen–antibody complexes (14, 15). In our cohort, ROC curve analysis further identified an optimal cutoff value of ≥17.47 RU/mL for predicting tissue PLA2R deposition (AUC = 0.851). Previous researches have demonstrated a strong correlation between serum anti-PLA2R antibody positivity and glomerular PLA2R antigen deposition (15–18). The 2021 KDIGO guidelines also recommend that in patients with detectable serum anti-PLA2R antibodies and clinical features consistent with MN, renal biopsy may be omitted (12). The cohort-derived anti-PLA2R antibody cutoff proposed in this study may support noninvasive diagnosis in this specific clinical context; however, it should be regarded as hypothesis-generating until externally validated. Reported diagnostic thresholds for PMN vary widely across studies, ranging from approximately 2 to 40 RU/mL (19, 20). Notably, a large Chinese multicenter observational study reported an “optimal” cutoff of 3.8 RU/mL (sensitivity 71%, specificity 90%); however, that study aimed to discriminate PMN from other glomerular diseases and therefore optimized the cutoff for clinical diagnosis (21). In contrast, our ROC analysis used biopsy-proven glomerular PLA2R antigen deposition as the reference standard, evaluating the ability of circulating antibodies to predict tissue PLA2R expression. Moreover, given the single-center retrospective design and the semi-quantitative, platform-dependent nature of RU/mL measurements—affected by calibration strategy, antigen presentation, and analytical sensitivity—substantial variability across studies is expected. Therefore, although the proposed cutoff may be useful for noninvasive diagnosis in this specific setting(namely in Chinese patients with typical nephrotic syndrome, no clinical evidence of secondary causes, and using the same ELISA platform), it should not be interpreted as a universal serological threshold and requires external validation in independent, multicenter Chinese cohorts prior to clinical application. Moreover, anti-PLA2R antibodies have also been reported in secondary and malignancy-associated MN. Therefore, positive serologic results should always be accompanied by comprehensive clinical evaluation to exclude secondary causes (22, 23).

Regarding the association between serum anti-PLA2R antibody titers and prognosis, no significant differences were observed in our cohort after risk stratification by baseline titer (low, medium, and high) (P = 0.573). This finding stands in contrast to several previous reports showing that higher anti-PLA2R antibody titers are associated with worse outcomes (24, 25). In our cohort, however, neither baseline anti-PLA2R seropositivity nor antibody titer was predictive of disease progression or time to partial remission (26). Such discrepancies may be attributed to study limitations, including single-center bias, lack of standardized sequential antibody monitoring during follow-up, and insufficient statistical power to detect moderate effects due to sample size constraints. Therefore, future studies should emphasize the prognostic value of serial antibody measurements. We also found that baseline anti-PLA2R antibody titers were positively correlated with 24-hour urinary protein excretion and dyslipidemia markers (including total cholesterol, triglycerides, and LDL-C), consistent with the findings of previous studies (27, 28). Existing evidence indicates that a decline in antibody titers is associated with clinical remission, whereas rising titers often predict worsening proteinuria during relapse. Dyslipidemia has also been identified as an independent risk factor for disease progression (29). These findings underscore the utility of serial anti-PLA2R antibody measurements as a dynamic biomarker reflecting both disease activity and metabolic sequelae. When combined with quantitative proteinuria and lipid monitoring, these parameters can improve the precision of individualized immunosuppressive strategies and enhance the accuracy of long-term renal survival prediction.

PLA2R-negative PMN patients exhibited distinct clinicopathologic characteristics, including a markedly higher proportion of females, better baseline renal function (higher eGFR and lower serum creatinine), elevated serum IgG levels, and, most notably, a significantly increased prevalence of C1q deposition (38.1% vs. 13.5%; *P* < 0.001). Early studies generally suggested that C1q deposition is uncommon in PMN and is typically associated with secondary MN, such as lupus nephritis (8, 30–32). Nevertheless, similar observations of aberrant C1q deposition have been reported in certain subgroups of PLA2R-negative PMN (9, 15). These findings may indicate complex pathogenic mechanisms: (i) Undetected secondary triggers (e.g., occult autoimmune/neoplastic disorders generating C1q-fixing immune complexes; (ii) Alternative-to-classical pathway crosstalk (e.g., C3 nephritic factors indirectly activating classical components, facilitating C1q co-deposition); or (iii) Innate complement activating properties of non-PLA2R antigens (e.g., cryptic epitope exposure in EXT1/2 or NELL-1 enhancing classical pathway initiation via C1q binding) (3, 32, 33). Collectively, this evidence suggests that C1q deposition does not exclude the diagnosis of primary PMN. However, vigilant monitoring for potential secondary etiologies remains warranted in PLA2R^-^/C1q^+^ patients.

In addition to the core antigens, this study also identified two rare antigens, THSD7A and NELL-1, while no positivity for EXT1, EXT2, or PCDH7 was detected in any patient. The prevalence of THSD7A antigen in renal tissue was 5.3%, consistent with previously reported rates ranging from 1% to 9% (34). Compared with THSD7A-negative patients, those positive for THSD7A were characterized by older age at onset, lower eGFR, and higher serum IgG levels, in line with prior reports (34, 35), suggesting distinctive clinical and pathological features associated with THSD7A positivity. Among PLA2R/THSD7A double-negative MN cases, one patient (5.9% of double-negative cases) was positive for NELL-1, a prevalence much lower than the 23% reported by the Mayo Clinic (36) and the 10% documented in studies from India (7). This discrepancy may be related to methodological differences (immunohistochemistry vs. mass spectrometry), the strict exclusion of secondary MN etiologies, or true racial and geographic variations in disease pathogenesis. The true prevalence of these rare antigens warrants further validation in large, multicenter cohorts using high-sensitivity detection methods such as mass spectrometry.

Of note, several studies have suggested that both THSD7A- and NELL-1-associated MN may be linked to malignancy. Tumor cells may ectopically express THSD7A or NELL-1, triggering autoantibody production and subsequent glomerular immune complex deposition (37, 38). The reported incidence of malignancy among THSD7A-positive MN patients ranges from 10–20% in Western populations (34, 39), whereas Asian cohorts have demonstrated lower rates, with no malignancies observed among the 9.1% (5/55) of patients who were THSD7A-positive (34), suggesting that geographic or ethnic factors may influence ascertainment or biology. For NELL-1-positive MN, malignancy rates are strikingly inconsistent—ranging from 0% in the Mayo Clinic series to as high as 4/5 in French studies (36), with prevalence estimates of 10–33% in elderly-predominant populations (8, 38). In our study, however, none of the seven THSD7A-positive patients nor the single NELL-1-positive patient developed malignancy at diagnosis or during a median follow-up of 62.9 months. Given the extremely low prevalence of these rare antigens, the statistical power of the present study is inherently limited, and the very small sample size severely restricts our ability to test the malignancy risks reported in previous literature. In addition, this retrospective study relied on routine clinical cancer evaluation rather than a standardized screening protocol, and the follow-up duration may still be insufficient to capture late-onset malignancies. Moreover, antigen detection based on IHC may be less sensitive than mass spectrometry–based approaches. Therefore, the findings regarding THSD7A- and NELL-1–associated malignancy should be regarded as descriptive observations only; the absence of observed events does not exclude a clinically meaningful association. Nevertheless, potential biological explanations, including differences in genetic background affecting immune recognition, regional cancer epidemiology, and environmental exposures that may modulate tumor risk and antigen-specific autoimmunity, remain worthy of further investigation. Notwithstanding the lower malignancy prevalence in Asians, routine age-appropriate screening is essential across all antigen subtypes, consistent with evidence from Western populations. Future prospective, multicenter studies incorporating standardized malignancy screening and harmonized antigen detection strategies (IHC combined with mass spectrometry) are required to clarify these associations.

After long-term follow-up (median 62.9 months), 42.4% of patients achieved CR and 36.4% achieved PR within 5 years, yielding an overall remission rate of 78.8%, while 21.2% showed no remission. In general, literature reviews suggest that under current treatment strategies, the 5-year total remission rate typically ranges from 60% to 85% (40). In our cohort, the 5-year renal survival rate was 81.95%, which is comparable to reported rate in Western cohorts with similar follow-up durations (75–89.4%) (10, 41). No significant differences were observed between PLA2R-positive and PLA2R-negative groups in response to immunosuppressive therapy or renal survival. Although some studies have suggested that PLA2R positivity may be modestly associated with remission, it does not independently determine long-term outcomes, including renal survival. This indicates that PLA2R positivity does not necessarily predict an unfavorable long-term prognosis (42, 43). Several limitations should be acknowledged when interpreting these long-term findings. The variable follow-up duration (interquartile range, 32.4–87.4 months) may have led to missed late events or relapses. The retrospective, single-center design, reliant on medical records and recall, introduces potential bias. Moreover, the absence of protocolized serial measurements of anti-PLA2R or other relevant antibodies during follow-up limits our ability to assess correlations between dynamic biomarker changes and long-term outcomes—such as sustained remission or late relapse—which are often more informative than a single baseline titer.

## Conclusion

In this extended (>5 years) Chinese cohort of PMN, we performed the first comprehensive screening of six podocyte target antigens. PLA2R remained the predominant antigen, whereas PLA2R-negative patients—mostly female and with frequent C1q deposition—showed distinct immunopathological patterns but similar long-term renal outcomes. We identified a cohort-specific anti-PLA2R antibody threshold (≥17.47 RU/mL) with good accuracy for predicting glomerular PLA2R deposition, supporting its potential role in noninvasive diagnosis within this specific clinical and ethnic context. However, this cutoff should be considered hypothesis-generating until validated externally. Serum anti-PLA2R antibody titers at baseline were not independently associated with long-term remission or renal survival, and overall prognosis was primarily driven by baseline proteinuria rather than antigen subtype. The detection rates of rare antigens such as THSD7A and NELL-1 were low, and conclusions regarding their association with malignancy risk remain limited. Collectively, these findings fill a major gap in long-term antigen-specific data for PMN, support antigen-guided, population-specific precision management of MN. Further multicenter, multi-omics studies are warranted to validate and refine these conclusions.

## Data Availability

The original contributions presented in this study are included in the article and/or supplementary material. Further inquiries or requests for raw data can be directed to the corresponding author.
