# Supplementary material for: Long-term outcomes in primary membranous nephropathy: a Chinese cohort study with novel target antigen
Source: Front Immunol. 2026 Mar 6;17:1761515. doi: 10.3389/fimmu.2026.1761515 (PMC13002824; doi:10.3389/fimmu.2026.1761515)

**Supplement references:**

[S1] Sethi S, Madden B, Debiec H, Morelle J, Charlesworth MC, Gross L, Negron V, Buob D, Chaudhry S, Jadoul M, Fervenza FC, Ronco P. Protocadherin 7-Associated Membranous Nephropathy. J Am Soc Nephrol. 2021 May 3;32(5):1249-1261. doi: 10.1681/ASN.2020081165. Epub 2021 Apr 8. PMID: 33833079; PMCID: PMC8259689.

[S2] Sethi S, Madden BJ, Debiec H, Charlesworth MC, Gross L, Ravindran A, Hummel AM, Specks U, Fervenza FC, Ronco P. Exostosin 1/Exostosin 2-Associated Membranous Nephropathy. J Am Soc Nephrol. 2019 Jun;30(6):1123-1136. doi: 10.1681/ASN.2018080852. Epub 2019 May 6. PMID: 31061139; PMCID: PMC6551791.

**Supplement Tables:**

Table S1. Baseline characteristics of censored vs non-censored patients

|  | **Censored（n=38）** | **Non-censored（n=94）** | **P** |
| --- | --- | --- | --- |
| Age | 50.00±12.77 | 50.61±12.92 | 0.8069 |
| Male (%) | 55.3 | 59.6 | 0.7949 |
| eGFR | 107.15（94.78-119.53） | 99.65（89.00-110.30） | 0.1616 |
| 24-hour urinary protein(g/24h) | 4.25（2.63-5.87） | 5.33（2.73-7.93） | 0.3017 |
| Albumin | 25.30（21.28-29.32） | 22.95（18.78-27.13） | 0.1222 |
| PLA2R-Ab | 15.09（1.78-126.88） | 42.27（2.36-148.42） | 0.1303 |
| C1q+ (%) | 13.5 | 17.1 | 0.6059 |
| Immunosuppression (%) | 86.8 | 81.9 | 0.4916 |

Table S2. Cox Proportional Hazards Regression Analysis Results of censored vs non-censored patients

|  | Adjusted HR | 95% CI | P-value |
| --- | --- | --- | --- |
| Censoring Only |  |  |  |
| Censored vs. Non-Censored | 0 | 1.0-Inf | P = 0.9964 |
| Adjusted for Covariates |  |  |  |
| Censored vs. Non-Censored | 0 | 1.0-Inf | P = 0.9964 |
| Age | 1.02 | 2.69-2.88 | 0.1720 |
| eGFR | 0.98 | 2.63-2.71 | P = 0.0237 |
| PLA2R Antibody (Positive vs. Negative) | 1.50 | 2.03-23.93 | P =0.2905 |

Table S3. Multivariable Cox regression of factors associated with renal survival in membranous nephropathy

|  | Adjusted HR | 95% CI | P-value |
| --- | --- | --- | --- |
| PLA2R antigen | 2.29 | 0.605~8.70 | P = 0.222 |
| Age | 1.02 | 0.983~1.06 | P = 0.279 |
| Male | 0.314 | 0.125~0.791 | P = 0.014 |
| eGFR | 0.983 | 0.964~1.00 | P = 0.105 |
| 24-hour urinary protien(g/24h) | 1.28 | 1.15~1.42 | P < 0.001 |
| C1q+ | 1.95 | 0.799~4.76 | P = 0.142 |
| Immunosuppression | 0.382 | 0.13~1.12 | P = 0.0797 |

**Supplement Figures:**

Figure S1. Negative IHC staining for PCDH7 in PMN


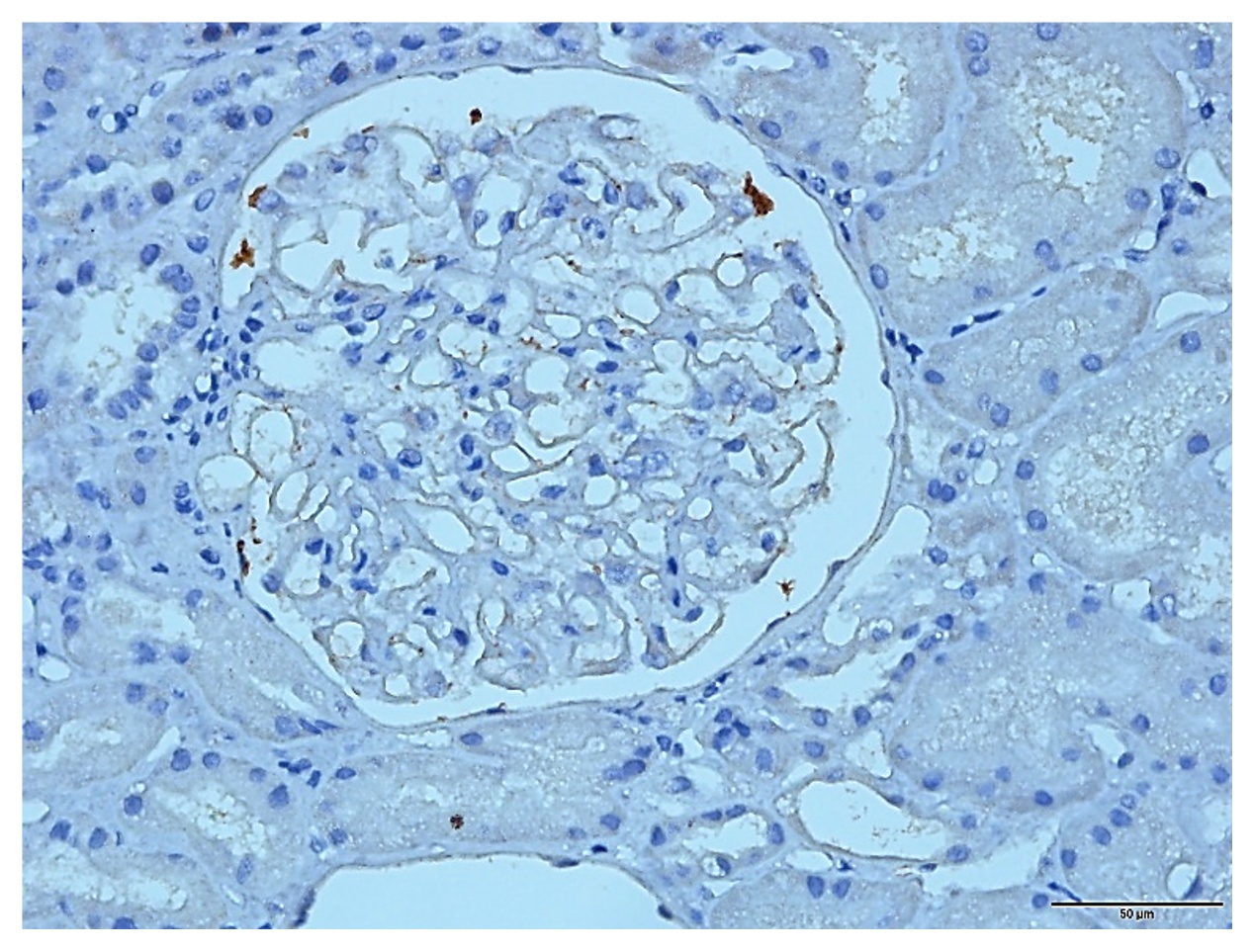


Figure S2. Negative IHC staining for EXT1 in PMN


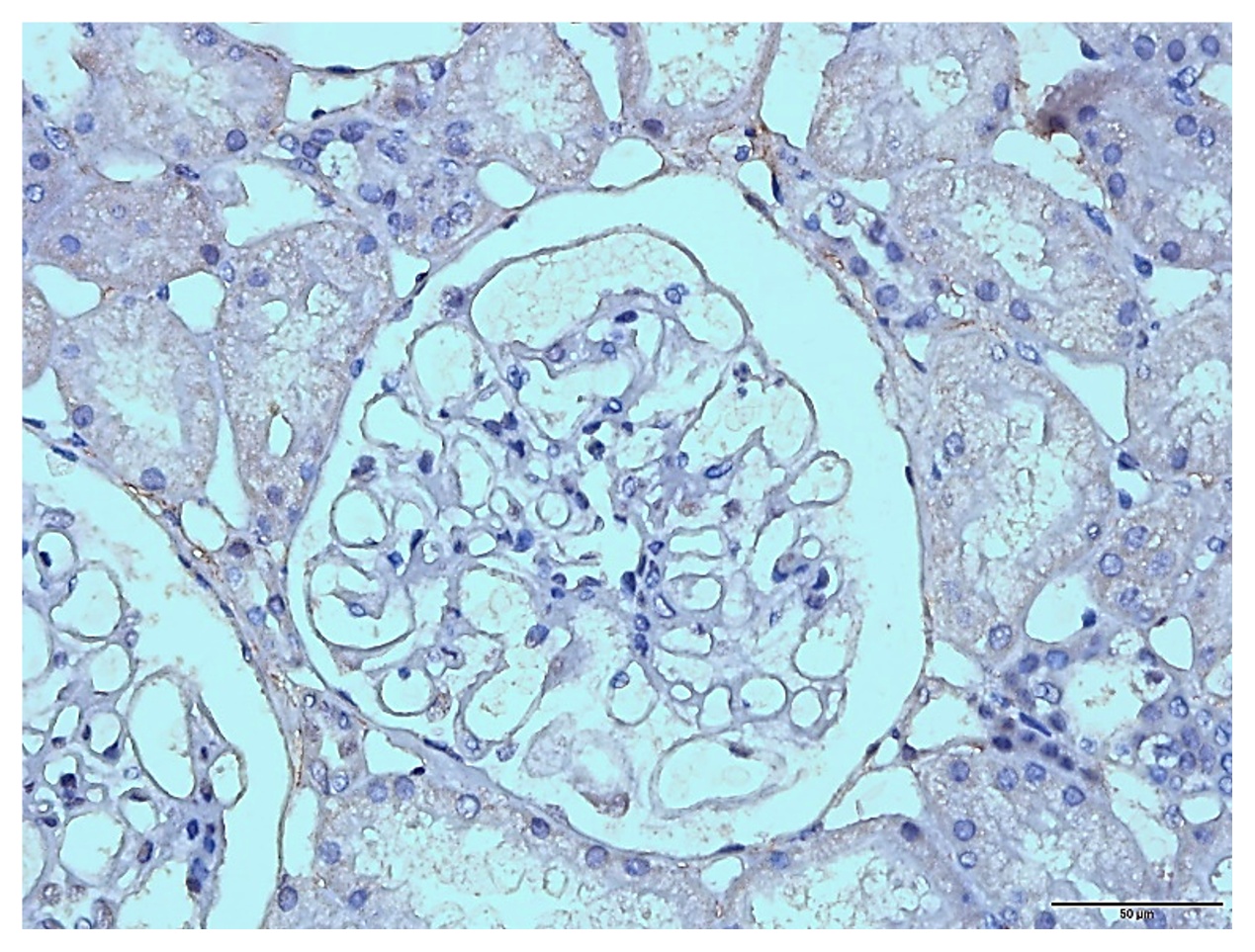
Figure S3. Negative IHC staining for EXT2 in PMN
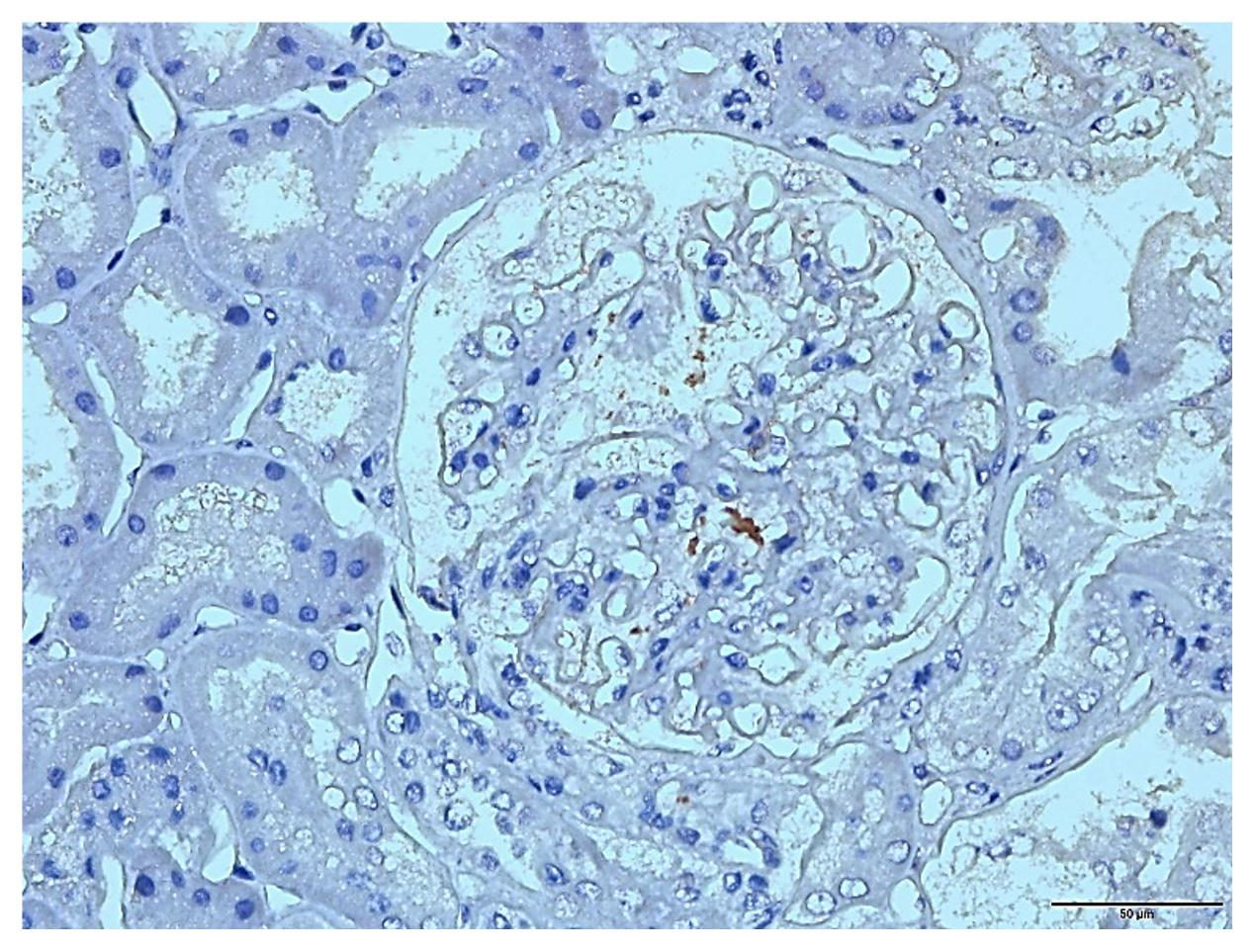


Figure S4 5-Year Renal Survival Estimated by Inverse Probability of Censoring Weighted (IPCW) Kaplan-Meier Analysis


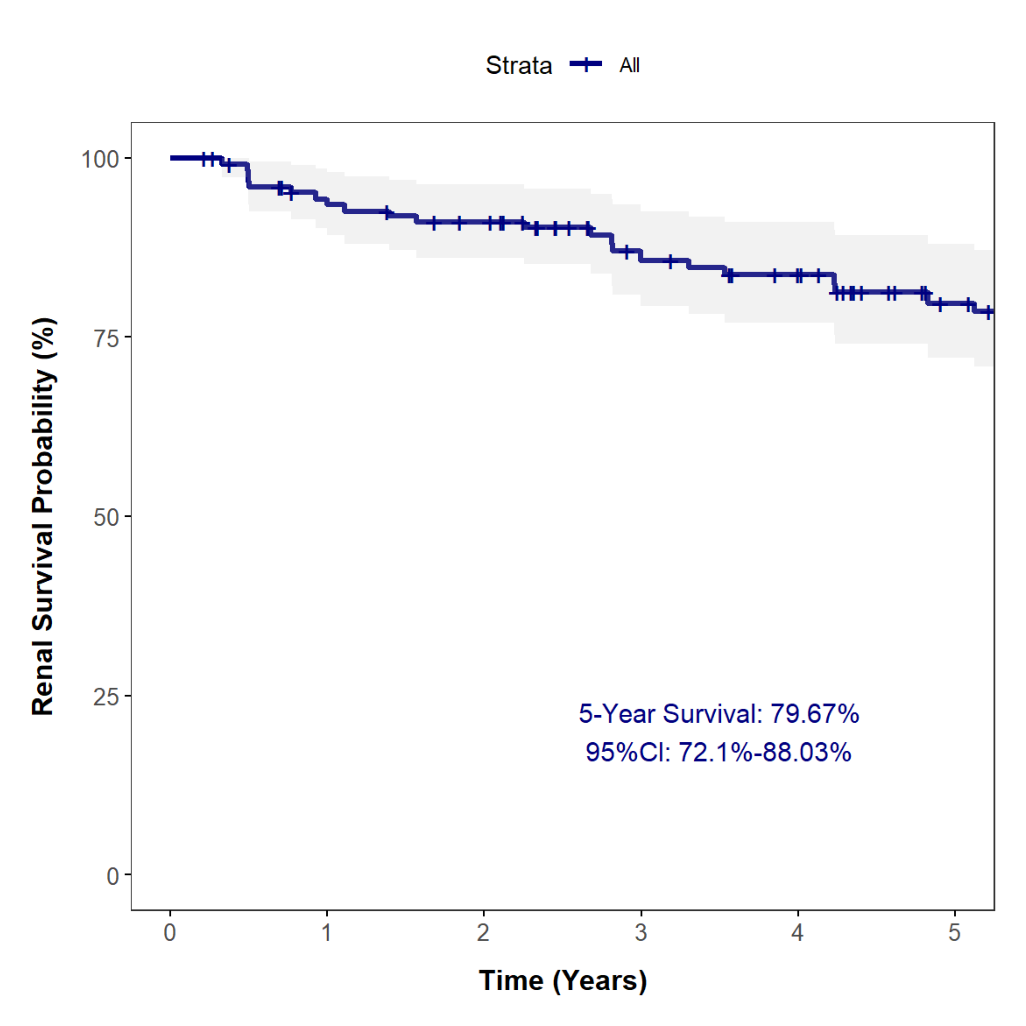


Figure S5 Kaplan-Meier Curve for PLA2R Antigen Positive vs Negative Groups


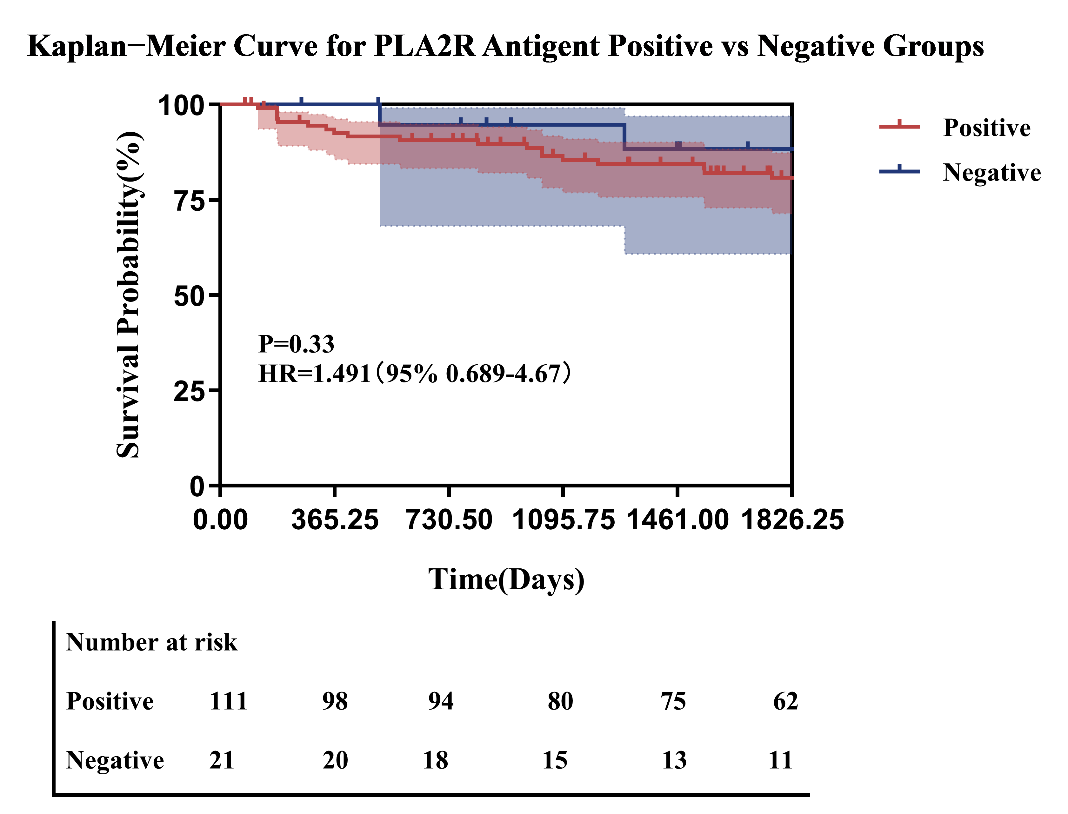

Supplement: Supplementary file 1 [file Table1.docx]
